# Effects of Phenytoin Therapy on Bispectral Index and Haemodynamic Changes Following Induction and Tracheal Intubation

**Published:** 2009-04

**Authors:** Parmod K Bithal, Mihir P Pandia, Rajendra S Chouhan, Hemanshu Prabhakar, Girija P Rath, Hari Hara Dash, Manish K Marda

**Affiliations:** 1 Professor, Department of Neuroanesthesia, All India Institute of Medical Sciences, New Delhi, India; 2 Assistant Professor, Department of Neuroanesthesia, All India Institute of Medical Sciences, New Delhi, India; 3 Associate Professor, Department of Neuroanesthesia, All India Institute of Medical Sciences, New Delhi, India; 4 Assistant Professor, Department of Neuroanesthesia, All India Institute of Medical Sciences, New Delhi, India; 5 Assistant Professor, Department of Neuroanesthesia, All India Institute of Medical Sciences, New Delhi, India; 6 Professor, Department of Neuroanesthesia, All India Institute of Medical Sciences, New Delhi, India; 7 Senior Resident, Department of Neuroanesthesia, All India Institute of Medical Sciences, New Delhi, India

**Keywords:** Phenytoin sodium, Anaesthetic requirement, Bispectral index, Intubation response

## Abstract

**Summary:**

Laryngoscopy and tracheal intubation (LTI) increase blood pressure and heart rate (HR). Intensity of these changes is influenced by the anaesthetic depth assessed by the bispectral index (BIS). We determined the effect of phenytoin on anaesthetic depth and its influence on haemodynamics following LTI. Fifty patients of ASA grades I and II on oral phenytoin 200 to 300mg per day for more than one week were compared with 48 control patients. Standard anaesthesia technique was followed. BIS, non invasive mean blood pressure (MBP) and HR were recorded 30, 60, 90 and 120 sec after LTI. Phenytoin group needed lesser thiopentone for induction, 5 mg (1.1) vs. 4.3 mg (0.7) [p=0.036]. BIS was significantly lower in the phenytoin group vs. the control 30, 60, 90 and 120 sec after LTI [43.1 (16.0) vs. 48.9 (14.9), p=0.068, 56.3 (16.7) vs. 64.3 (14.4), p=0.013, 59.8 (15.8) vs. 67.5 (12.1), p=0.008, 62.6 (14) vs. 68.9 (11.2), p=0.017, and 64.2 (11.3) vs. 69 (11.7), p=0.033], respectively. MBP was also lower in the phenytoin group 30, 60, 90 and 120 sec after LTI [112.8 mmHg (13.8), vs. 117.9 mmHg (18) p=0.013, 108.6 (12.8) vs. 117.5 (16) p=0.003, 106.1 mmHg (14.1) vs. 113.2 mmHg (14.9), p=0.017, 101.8 mmHg (13.8) vs. 109.5 mmHg (14.1), p=0.007], respectively. HR was lower in phenytoin group at 30 sec. (p=0.027), 60 sec (p=0.219), and again at 120 sec (p=0.022). Oral phenytoin therapy for over a week results in greater anaesthetic depth as observed using BIS, which also attenuated haemodynamic response of LTI.

## Introduction

Laryngoscopy and tracheal intubation (LTI) maneouvers induce undesirable hypertension and tachycardia.^1^ A significant factor that determines the severity of these haemodynamic perturbations is anaesthetic depth at this stage.^2^ Introduction of bispectral index (BIS) monitor into anaesthesia practice has made monitoring of anaesthetic depth feasible. Previous studies have demonstrated that noxious stimulation of laryngoscopy and tracheal intubation is associated with increases in BIS besides increases in blood pressure and heart rate.^3^ Therefore it is common practice to administer a variety of pharmacological adjunct either with premedication or at the time of induction of anaesthesia, to obtund/attenuate increases in BIS value as well as haemodynamics following laryngoscopy and tracheal intubation.^4^,^5^ Patients of intracranial tumours usually receive oral phenytoin from the day they are examined by the neurosurgeon to prevent seizures with their attendant complications. The commonly followed regimen in an adult patient is administration of phenytoin sodium 200 to 300 mg orally, at night. This dose is continued for varying duration in the postoperative period. At our centre, on the morning of surgery, these patients receive 100 mg phenytoin intravenous, approximately 90 min before induction of anaesthesia. It has been stated that a steady state of plasma concentration is achieved after at least one week of continuous oral phenytoin treatment. We hypothesized that because of its effects on the central nervous system and cardiovascular system, phenytoin therapy could influence the BIS value and haemodynamic responses during induction of general anaesthesia and tracheal intubation. The objectives of the present study were to compare the BIS value and haemodynamic responses associated with induction and tracheal intubation in patients with or without phenytoin therapy.

## Methods

This preliminary observational study was approved by local Ethics committee and informed consent for participation in the study was obtained from 120 adult patients of either gender and American Society of Anesthesiologists (ASA) physical status I and II, scheduled for elective intracranial and spine surgery. Patients of intracranial surgical procedures, who were taking oral phenytoin 200 to 300 mg daily for more than seven days, were included in the study. Exclusion criteria were patients with intracranial vascular lesions, patients on phenytoin for less than one week, altered sensorium, cardiovascular, metabolic or chronic obstructive pulmonary disease, drug/alcohol abuse, and anticipated difficult laryngoscopy/intubation. They were divided into two groups: patients on phenytoin were taken as phenytoin group while patients of spine surgery not on phenytoin therapy served as control. All patients were premedicated with 5 mg oral diazepam 2 hours before anaesthesia induction. Patients in the phenytoin group received an IV bolus of 100 mg phenytoin approximately 90 min before induction, as per our hospital protocol. In the operation theatre BIS sensors were attached as per instructions of the manufacturers and connected to Aspect-2000 BIS monitor (Aspect medical system, Natick, MA, USA). Routine monitoring consisted of ECG, heart rate (HR), mean blood pressure (MBP) and SpO2 (Datex Engstrom, Helsinki, Finland). Non-invasive blood pressure (NIBP) monitor was set on stat mode which displays and records blood pressure reading every 30 sec. BIS smoothing rate was set at 15 sec. After noting baseline values of BIS, mean blood pressure (MBP) and HR, patients were administered fentanyl 2mcg.kg^−1^ approximately, and five minutes later thiopentone was administered until abolition of eye-lash reflex. Rocuronium 1mg.kg^−1^ was administered and trachea intubated 90 sec later. BIS, MBP and HR were recorded after induction (immediate prior to laryngoscopy) and subsequently, at 30 sec interval for 120 sec after tracheal intubation. Anaesthesia during the study period was maintained with 66% nitrous oxide in oxygen, and lungs were ventilated mechanically. The study was terminated at this point and subsequent anaesthesia proceeded according to the surgical requirements. The time to intubation was taken from introduction of laryngoscope to tracheal tube placement. Patients in whom more than one attempt or more than 15sec were required for tracheal intubation were excluded from the study.

Statistical analysis: Data are expressed as mean ± SD. Statistical analysis within each group was performed with repeated measures analysis of variance (ANOVA) test and where significance was observed, Bonferroni test was applied for comparison with baseline values. Independent sample ‘t’ test was used to compare numerical data between two groups. P value less than 0.05 was inferred as significant.

## Results

A total of 120 patients were enrolled in the study, of which 22 (12 from control and 10 from phenytoin group) were excluded, either because time taken for intubation exceeded 15sec or multiple attempts were needed for intubation. Therefore, data were analyzed from 98 patients (48 of control and 50 of phenytoin group). Two groups were comparable in respect of age, sex and weight (Table 1). Patients of the phenytoin group lost eye-lash reflex at a significantly smaller dose of thiopentone compared with the control group. Time taken to intubate trachea was comparable in the two groups (Table 2).

**Table 1:** Demographic data of patients [Mean ± SD or number]

	Phenytoin group (n = 50)	Control group (n = 48)	p value
Age (years)	36.6 ± 10.4	38.6 ± 11.6	NS
Weight (Kg)	56.5 ± 11.1	59.8 ± 11.8	NS
M:F	27:23	27:21	NS

n– number of patients in each group

M:F – male: female, NS – not significant

**Table 2:** Anaesthetic requirement and time for tracheal intubation during induction of general anaesthesia. [Mean ± SD]

	Phenytoin group (n = 50)	Control group (n = 48)
Total dose of thiopentone sodium (mg/kg)	4.3 ± 0.7*	5.0 ± 1.1
Tracheal intubation time (seconds)	12.2 ± 2.9	12.5 ± 2.6

* p < 0.05 as compared to the control group, n – number of patients in each group

BIS: Baseline BIS was comparable in two groups. Compared with baseline it decreased significantly in both groups in response to thiopentone administration, the decrease being comparable in two groups. In both groups tracheal intubation resulted in significant increases in BIS which persisted throughout the study duration. Greatest increases in BIS were observed 120 sec post intubation in both the groups. The increases in BIS were significantly less pronounced in the phenytoin group, at each time point (Table 3).

**Table 3:** Bispectral index values at various time intervals following induction of general anaesthesia and tracheal intubation. [Mean ± SD]

	Phenytoin group (n = 50)	Control group (n = 48)	p value (Inter-group)
Baseline	94.9 ± 5.7	96.7 ± 3.9	0.08
Immediately after induction	43.1 ± 16.0*	48.9 ± 14.9*	0.06
30 sec after intubation	56.3 ± 16.7*	64.3 ± 14.4*	0.01
60 sec after intubation	59.8 ± 15.7*	67.5 ± 12.1*	0.008
90 sec after intubation	62.6 ± 14.0*	68.9 ± 11.2*	0.01
120 sec after intubation	64.2 ± 11.3*	69 ± 10.7*	0.03

* p < 0.001 as compared to the baseline values in the respective group, n – number of patients in each group

MBP: Baseline MBP was comparable in two groups. Thiopentone administration produced no alteration in MBP in either group. Tracheal intubation increased MBP in both the groups at 30, 60 and 90 sec. compared with baseline. Maximal increases in MBP in both groups were recorded 30 sec post tracheal intubation. The increases in MBP were significantly lesser in the phenytoin group (Table 4).

**Table 4:** Mean blood pressure values(mmHg) at various time intervals following induction of general anaesthesia and tracheal intubation[Mean ± SD]

	Phenytoin group (n = 50)	Control group (n = 48)	p value (Inter-group)
Baseline	99 ± 10.9	103.5 ± 11.3	0.05
Immediately after induction	92.5 ± 12.2	92.8 ± 13.4	0.28
30 sec after intubation	112.8 ± 13.8**	117.9 ± 18.0#	0.01
60 sec after intubation	108.6 ± 12.8**	117.5 ± 16.0#	0.003
90 sec after intubation	106.1 ± 14.1*	113.2 ± 14.9#	0.01
120 sec after intubation	101.8 ± 13.8	109.5 ± 14.1	0.007

* p < 0.05 as compared to the baseline values in the respective group

** p < 0.01 as compared to the baseline values in the respective group

# p < 0.001 as compared to the baseline values in the respective group, n – number of patients in each group

HR: Baseline HR was similar in two groups. There were significant increases of HR in both groups in response to thiopentone administration and tracheal intubation compared with baseline throughout the study period. Maximal increases in HR were observed 30 sec post intubation in both groups. The magnitude of increases of HR was significantly lesser in the phenytoin group at each time point, except 90 sec post intubation, when it was comparable in both groups (Table 5).

**Table 5:** Mean heart rate values(bpm) at various time intervals following induction of general anaesthesia and tracheal intubation. [Mean ± SD]

	Phenytoin group (n = 50)	Control group (n = 48)	p value (Inter-group)
Baseline	82.7 ± 16.5	87.3.5 ± 18.3	0.18
Immediately after induction	96.1 ± 16.2**	102.3 ± 13.6**	0.04
30 sec after intubation	114.4 ± 18.3**	122.1 ± 15.6**	0.02
60 sec after intubation	111.1 ± 9.0**	119.1 ± 13.8**	0.01
90 sec after intubation	109.8 ± 18.0**	115.9 ± 13.3**	0.06
120 sec after intubation	105.8 ± 22.3*	114.5 ± 13.6**	0.02

* p < 0.01 as compared to the baseline values in the respective group

** p < 0.001 as compared to the baseline values in the respective group, n – number of patients in each group

## Discussion

Induction of anaesthesia expectedly decreased BIS in both the groups but the impact on BIS was greater in the phenytoin group, presumably because of potentiation of hypnotic effect of thiopentone by phenytoin. A significant smaller dose of thiopentone required to abolish eye-lash reflex in the phenytoin group gives credence to this potentiation theory. Previous studies have demonstrated that tracheal intubation is associated with increase in BIS value.^3^,^6^ In agreement with those studies, we too observed increase in BIS values following tracheal intubation in both the groups but the increase was less marked in the phenytoin group compared to the control group throughout the study period. We excluded patients in whom laryngoscopy and/or tracheal intubation required more than one attempt or when the duration of LTI exceeded 15 sec. The procedure of tracheal intubation is usually accomplished within 10 to 15 sec, while duration longer than this denotes difficult airway which might alter anaesthetic depth, thereby, our observations.

Earlier studies by Turner et al,^2^ Stoelting,^7^ and Bucx et al^8^ have demonstrated that degree of reflex response to laryngeal stimulation appears to vary with the depth of anaesthesia, the duration and difficulties encountered during laryngoscopy and tracheal intubation. Guignard and colleagues^9^ reported that maximal increase in BIS value as well as haemodynamic values occurred during first 120 sec following orotracheal intubation, therefore we limited our observations to 120 sec post intubation. Our observations on BIS following laryngoscopy and tracheal intubation further strengthen our hypothesis that in the phenytoin group a greater depth of anaesthesia was responsible for a smaller increase in BIS value compared to the control group in spite of significantly smaller dose of thiopentone required for induction of anaesthesia in the former group. BIS is a predictor of depth of anaesthesia/hypnosis and arousal reaction during induction of anaesthesia and tracheal intubation. An attenuated BIS increase in the phenytoin group reflects a greater suppression of cortical activity and thereby, the level of consciousness. Increased gamma amino butyric acid (GABA) in brain may also result in deeper plains of anaesthesia. However, at normal phenytoin concentrations no changes of spontaneous activity or response to iontophoretically applied GABA are detected. At concentrations five to ten folds higher, multiple effects of phenytoin are evident including reduction of spontaneous activity and enhancement of response to GABA.^10^ Therefore, it is unlikely that GABA played any role in increasing the anaesthetic depth in the phenytoin group. Laryngoscopy and tracheal intubation are known to increase blood catecholamine levels.^11^ The importance of adrenergic system for the modulation of consciousness has been known since long.^12^ Previous studies have demonstrated that exogenously administered adrenaline causes clinical signs of arousal associated with an increase in BIS in deeply sedated patients^13^ but not in presence of deep anaesthesia.^14^ Furthermore, Hirota and colleagues have demonstrated that BIS levels are significantly correlated with plasma nore-pinephrine concentrations after oral diazepam premedication.^15^ Thus one more reason of relatively smaller increase in BIS in the phenytoin group could be the blunting of the action of endogenously released catecholamines on the cerebral cortex or suppression of release of catecholamines during LTI since the intensity of reflex response to laryngeal stimulation tends to vary with the depth of anaesthesia. The same factors were probably responsible for a smaller increase in BIS in the phenytoin group over the next two minutes post tracheal intubation.

Many drugs, including the recently reported gabapentin^16^ and landiolol,^17^ have been shown to be effective in attenuating haemodynamic responses associated with LTI in healthy patients. Even deepening of anaesthesia has also been recommended to blunt these responses.^18^ Importance of avoidance of blood pressure fluctuations during anaesthesia induction, and tracheal intubation cannot be overemphasized. Abrupt increase in BP during tracheal intubation has been reported to cause myocardial ischaemia,^19^ and rupture of intracranial aneurysm in susceptible patients.^20^ Since tachycardia appears to be associated more frequently with myocardial ischaemia than does hypertension, most common approach towards blunting haemodynamic response to laryngoscopy and tracheal intubation is use of beta-adrenergic antagonist.^21^ However, excessive negative chronotropic and inotropic action of beta blockers may be harmful in susceptible cardiac patients.^22^ We have no explanation for the exact mechanism by which phenytoin attenuates the pressor responses of laryngoscopy and tracheal intubation but as mentioned above it may do so by increasing the anaesthetic depth and thereby, resulting in suppression of endogenous catecholamine release. Stability of haemodynamic variables in the phenytoin group suggests that these patients blunt the insult of laryngoscopy and tracheal intubation better.

Phenytoin is absorbed slowly by oral route and a steady state of plasma concentration is achieved after at least one week of continuous treatment.^23^ For this reason we included patients who were taking this medicine for more than a week. Oral administration of phenytoin generally causes few side effects unless the patient has severe underlying cardiac disease. Intravenous administration at a rate exceeding 50 mg/min can have potentially catastrophic haemodynamic results.^24^ It has stabilizing effect on neuronal membrane by inhibiting voltage sensitive sodium channels. This affects both the nerves and also the cardiac muscles. It inhibits seizure activity without causing generalized central nervous system depression.

There are certain limitations of our study. The design of the study did not permit us blinding, thereby, enhancing the chances of bias in our observations. We avoided volatile agents over the study period to prevent their influence on haemodynamic and BIS value because different patient population in two groups might respond differently to these agents. Since maximal haemodynamic and BIS changes are observed within the first 120 sec after intubation we didn't deem it necessary to continue the study beyond this period.

In conclusion, patients on oral phenytoin 200 to 300 mg, daily dose for more than 7 days and who were also given 100mg of the drug intravenously, 90 min prior to anaesthesia induction, require a relatively lesser dose of thiopentone for induction of anaesthesia compared with the controls. Moreover, because of greater anaesthetic depth (as seen by BIS monitoring) the haemodynamic insult of LTI is blunted in them. Whether a single oral/intravenous dose administered on the day of surgery would attenuate the haemodynamic response to laryngoscopy and tracheal intubation requires further evaluation.
